# Advancing Research on Racial–Ethnic Health Disparities: Improving Measurement Equivalence in Studies with Diverse Samples

**DOI:** 10.3389/fpubh.2014.00282

**Published:** 2014-12-22

**Authors:** Hope Landrine, Irma Corral

**Affiliations:** ^1^Center for Health Disparities, Brody School of Medicine, East Carolina University, Greenville, NC, USA; ^2^Department of Psychiatry and Behavioral Medicine, Brody School of Medicine, East Carolina University, Greenville, NC, USA

**Keywords:** health disparities, race–ethnicity, measurement equivalence, scaling, methods

## Abstract

To conduct meaningful, epidemiologic research on racial–ethnic health disparities, racial–ethnic samples must be rendered equivalent on other social status and contextual variables via statistical controls of those extraneous factors. The racial–ethnic groups must also be equally familiar with and have similar responses to the methods and measures used to collect health data, must have equal opportunity to participate in the research, and must be equally representative of their respective populations. In the absence of such measurement equivalence, studies of racial–ethnic health disparities are confounded by a plethora of unmeasured, uncontrolled correlates of race–ethnicity. Those correlates render the samples, methods, and measures incomparable across racial–ethnic groups, and diminish the ability to attribute health differences discovered to race–ethnicity vs. to its correlates. This paper reviews the non-equivalent yet normative samples, methodologies and measures used in epidemiologic studies of racial–ethnic health disparities, and provides concrete suggestions for improving sample, method, and scalar measurement equivalence.

## Introduction

The term *health disparities* refers to patterns of health that mirror patterns of social status. Health disparities exist when those who occupy high social status positions enjoy superior health, while those who occupy low social status positions suffer inferior health (1–4). *Social status* refers to a socially defined group’s position (rank) in a hierarchical (stratified) society in terms of their power (possession and control of goods and resources), privilege (access to said goods and resources), and prestige [social-moral evaluation, with some people viewed as “better” than others (5–7)]. The social status hierarchies examined in health disparities research in the United States include those based on race–ethnicity, socioeconomic status (SES) position, and other factors (1). Within these hierarchies, those who occupy low status positions [e.g., racial–ethnic minorities (REMs), the low-SES] exhibit significantly poorer health than their higher-status counterparts (1–4). There are well-known racial–ethnic and SES disparities in cardiovascular disease (8), diabetes (9), asthma (10), and cancer (11), as well as in obesity, mortality, self-rated health, birth outcomes, health behaviors, and other aspects of health (12).

Epidemiologic studies compare the health of high and low status groups and supply the basic data (e.g., above) on health disparities. Those data shape population forecasts, motivate new programs and policies, and guide the allocation of resources and the evaluation of health services. In short, epidemiologic studies of health disparities are fundamental to plans to reduce disparities and are the evidence of progress in so doing (13, 14).

### The importance of measurement equivalence

To conduct meaningful studies of health disparities, the social status groups compared (e.g., racial–ethnic groups) must be rendered equal on other status and social-contextual variables via statistical controls of such extraneous factors. Likewise, all status groups must have similar responses to the methods and measures, must have equal opportunity to participate in the research, and must be equally representative of their populations. In the absence of such *measurement equivalence*, group comparisons are confounded by a variety of variables that are correlated with group-membership, and render findings uninterpretable (15–17); group differences in health cannot be attributed to group-membership if the samples, methods, and measures are incomparable across groups because of uncontrolled, correlated factors (15–17).

Thus, the problem of measurement equivalence in health disparities research “is not an esoteric, psychometric issue that has little or no consequences for science, policy, or medicine,” [(15), p. S205)]. Instead, measurement equivalence is fundamental to knowledge of health disparities, and to scientific, policy, and public opinions and decisions based on that knowledge (2). This paper explains and provides detailed examples of the lack of measurement equivalence in health disparities research conducted in the US, and presents concrete strategies for improving equivalence. Because of space limitations, we address *Sample*, *Method*, and *Scalar* equivalence only, and omit the many other types of equivalence (18–20). Likewise, space permits us to address these three forms of equivalence only for REMs (i.e., African-, Latino-, Asian-, and Native Americans). We emphasize, however, that the problems of measurement equivalence in studies of racial–ethnic health disparities apply to studies of health disparities among all other social status groups (e.g., gender, SES groups).

## Sample Equivalence

Sample equivalence exists when the status groups examined have been rendered equivalent on social, contextual, and other important correlates of race–ethnicity via statistical control of those correlates (21–24). Studies of racial–ethnic health disparities compare the health of REMs to that of whites, and attribute the differences found to race–ethnicity (i.e., they report racial–ethnic disparities) in the absence of evidence that race–ethnicity, rather than its many correlates, accounts for the differences, i.e., in the absence of evidence of sample equivalence (15–24).

### Correlates of race–ethnicity

Racial–ethnic groups differ on numerous social, cultural, and contextual variables that are relevant to health, but these usually are not measured (or are not measured adequately) and controlled in epidemiologic studies. Three of the many known correlates of race–ethnicity are SES (21–24), residential segregation [e.g., Ref. (25–29)], and numerous cultural variables (discussed later here). These correlates are universal in that they apply to all racial–ethnic groups, including whites. In the absence of control of these and other known correlates of race–ethnicity, racial–ethnic differences in health cannot be attributed to race–ethnicity rather than to its correlates with reasonable certainty.

Among the many known correlates of race–ethnicity, social-demographic variables are most often measured in epidemiology. The focus on such correlates (e.g., income, education) reflects a narrow understanding of the nature and production of racial–ethnic hierarchies in the US, and diverts attention from the health-relevant but usually unmeasured variables (e.g., segregation, discrimination) that maintain and are the core of racial–ethnic inequality (30–33). Likewise, the focus on cultural–demographic correlates (e.g., nativity, language) reflects a similarly narrow view of the nature and survival of REM cultures in a white-dominated society, and ignores the complexities of acculturation (adaptation) involved (31–33). Moreover, the range of social- and cultural–demographic correlates measured is itself narrow, with many important variables usually excluded.

For example, possessing a landline telephone (discussed later) is one social–demographic correlate of race–ethnicity (and SES) that contributes to health but is not measured in racial–ethnic health disparities research. Wage theft is another social–demographic correlate that also contributes to health (34). Wage theft refers to being paid less than the mandatory minimum wage, non-payment of overtime, refusal of meal and other breaks, confiscation of tips, pay deductions for being sick and for taking meals, and other illegal but common employment practices (34). Low-SES REMs are significantly more likely than other groups to be subjected to wage theft (34), yet neither wage theft nor (its associated) hazardous-working conditions is measured and controlled in normative studies of racial–ethnic health disparities. Similarly, religion is merely one of many cultural–demographic correlates of race–ethnicity that contributes to health: Members of the same racial–ethnic group who participate in different religions differ significantly in their health status and health behaviors (35), yet religion rarely is included in studies of racial–ethnic health disparities.

Moreover, many other correlates of race–ethnicity remain unknown. Indeed, the research of race–ethnicity scholars in anthropology, sociology, and other disciplines [e.g., Ref. (2–7, 30–33)] in part consists of the ongoing discovery of new correlates and processes of race–ethnicity. Because these unknown correlates cannot be included in studies, the correlates that epidemiologists measure and control cannot be assumed to be sufficient to render racial–ethnic samples equivalent.

Lack of adequate measurement and control of the known correlates of race–ethnicity, and the presence of unmeasured, unknown or newly discovered correlates together mean that *racial–ethnic samples in studies of health disparities are by definition non-equivalent (incomparable)*. Strategies for improving racial–ethnic sample equivalence can be used, however, and enhance tentative attributions of racial–ethnic health differences to race–ethnicity. The two examples below illustrate the problems of racial–ethnic sample non-equivalence and provide concrete suggestions for improving it.

#### Measuring and controlling SES to improve racial–ethnic sample equivalence

All racial–ethnic groups occupy SES positions, and those positions contribute to their health (8–12, 21). Thus, epidemiologists often (but not always) measure and control SES in studies of racial–ethnic health disparities (21, 36–40). The normative practice for the past several decades has been to measure SES as household income, and (less often) as education and occupation as well. Income is by far the most frequently used measure of SES in US research (21, 24, 37–40). This is evident in the Behavioral Risk Factor Surveillance System (BRFSS), the National Health Interview Survey (NHIS), the National Health and Nutrition Examination Survey (NHANES), and other population studies in which income is measured in many different ways (39).

As shown in Table 1, there are large, statistically significant, racial–ethnic differences in income. Such differences hold across levels of education and occupation (2, 6, 33, 34, 41, 42); because of employment discrimination, REMs have significantly lower incomes than whites of the same education (39) and same occupation (41, 42). After controlling for income, racial–ethnic health disparities invariably remain. There is substantial variation in health unexplained by income, and the normative practice has been to attribute that to race–ethnicity (21, 24, 36–40). The problem with doing so is that income is an inadequate measure of the meaning and complexity of SES by race–ethnicity, and is non-equivalent across racial–ethnic groups (21–24, 37–40), in the five ways summarized below.

**Table 1:** **Median and mean household income (43) and household size (44) by Race–Ethnicity, 2012**.

	Median	Mean	Mean household size
Whites^a^	57,009	77,834	2.38
African-Americans	33,718	48,160	2.55
Latinos	39,005	53,422	3.36
Asians^b^	68,636	91,400	2.98

*^a^Excludes White Hispanics/Latinos*.

*^b^Excludes Pacific-Islanders*.

##### Household size

Income does not take household size into account (43, 44). An income of $30,000 for a one-person household is usually treated as the same as an income of $30,000 for a five-person household even though the larger household has higher expenses and is poorer; the same income is non-equivalent across household size. Studies that measure SES as income rarely control for household size, yet the household sizes of REMs often are significantly larger than those of whites [Table 1; (44)]. Controlling for income without controlling for household size underestimates income differences between REMs and whites, and does not control for income. REMs not only have lower incomes but also often support more people with them.

##### Housing discrimination

One manifestation of pervasive housing discrimination is that the cost of housing (renting or buying) is significantly higher for REMs than for whites (45–51). REMs are twice as likely as whites to spend 50% or more of their incomes on housing alone (37, 45–51). Consequently, REMs have less money available for food, utilities and other essentials, and for savings accounts than whites of matched income (37, 45–51). The same income does not go as far for REMs as it does for whites, meaning that income is non-equivalent across race–ethnicity (37, 45–51). The significantly higher cost of housing for REMs results in greater financial hardships among them than among whites of the same income (37), and these (and lack thereof) are one aspect of the lived experience of SES in the US. The higher cost of housing for REMs also has been shown to contribute causally to their higher rates of poverty (47).

##### Credit and retail discrimination

Moreover, as a result widespread racial–ethnic discrimination, REMs also pay significantly more than whites for goods and services such as cars, car insurance, home loans, and other forms of credit, and for groceries, gasoline, and water and sewer services in their neighborhoods [e.g., Ref. (52–57)]. The same income involves lower ability to meet basic needs (and save money) among REMs than whites. This example further underscores the non-equivalence of income across racial–ethnic groups.

##### Single time-point measures

In addition, income is unreliable because it fluctuates considerably during the year. More than 50% of the US population experiences a significant change (an increase or decrease of ≥30%) in income during a year, with such changes more likely for REMs and low-SES groups (37). This means that the single time-point (cross-sectional) measure of income commonly used reflects that time-point only, and overlooks periodic poverty among REMs (36, 58, 59). Moreover, although income fluctuates during the year, it also tends to be somewhat stable over the lifetime (58–63). REMs are more likely than whites to experience long-term and lifetime exposure to financial disadvantage and poverty at the household and neighborhood levels (58–63). Such long-term exposure has greater negative impacts on health than short-term exposure, and accounts for variance in racial–ethnic health disparities that short-term exposures do not (38, 58–63). Cross-sectional measures of income thereby underestimate the contribution of income to racial–ethnic health disparities (58–63).

##### Non-response bias and income imputation

Finally, non-response rates to income questions are notoriously high, with REMs (African-Americans in particular) most likely to be non-responders (37, 64). Consequently, response bias characterizes income data from REMs insofar as the small percentage of REM responders are likely to differ in health (and other factors) from the majority of REM non-responders. Because of their high non-response rates, income often is imputed for substantial percentages of REMs; such imputations can yield inaccurate (i.e., higher) estimates of REM income (36, 64) and thereby underestimate the role of income in racial–ethnic health disparities.

Income is not the only normative measure of SES that is non-equivalent across racial–ethnic groups in terms of money available for basic needs and exposure to disadvantage (21–24, 37–40, 58–63). Education and occupation present similar problems: REMs who have the same education (39) and identical occupation (41, 42) as whites receive significantly lower incomes and have poorer life circumstances, such that neither education nor occupation is equivalent across race–ethnicity (21–24, 37–42, 58–63). Thus, controlling for income and education and occupation does not control for SES (21–24, 37–40, 58–63). Attributing racial–ethnic health disparities to race–ethnicity rather than to SES because one has controlled for these normative, non-equivalent SES measures is premature at best.

#### Alternatives to income

Many alternative measures of SES that improve SES measurement equivalence have been proposed. These facilitate controlling for SES, and enhance racial–ethnic sample equivalence to permit tentative attributions of health differences to race–ethnicity. Three of the many alternatives to income are equivalence-adjusted income, wealth, and area-based SES.

##### Equivalence-adjusted income

Equivalence-adjusted income is an income measure that takes household size into account (43), and thereby controls for racial–ethnic differences on that variable (Table 1). An equivalence-adjusted income of $30,000 for one person is more than twice that of an equivalence-adjusted income of $30,000 for a four-person household of two adults and two children [(43), p. 9–10]. Data on equivalence-adjusted income and how it is calculated by the U.S. Census Bureau are presented elsewhere (43). Although this measure is superior to income as a measure of SES, it is rarely used in health disparities research. Moreover, equivalence-adjusted income does not resolve the non-equivalence of income in terms of the higher housing and other prices that REMs pay (55).

##### Wealth

Wealth refers to financial resources such as property (homes), savings accounts, stocks, cars, and other assets (65–67). Racial–ethnic differences in wealth are several magnitudes larger than differences in income (65–67); whites have significantly greater wealth (net worth) than REMs of matched income (65). The median, overall wealth of whites ($111,740) is 15 times higher than that of African-Americans ($7,113) and 13 times higher than that of Latinos [$8,113; (65), p. 3].

Home ownership is the key component of wealth and plays an important role in financial security, i.e., in the lived experience of SES (65–67). As shown in Table 2, 98% of whites own their place of residence, compared to less than 60% of Asians and less than 45% of African-Americans and Latinos. Home ownership alone reveals that whites have significantly higher SES than all REMs, independent of income. In addition, significantly greater percentages of REMs than of whites lost home-value (Table 2) during the housing-market crash of 2005–2010 (65). This is because larger percentages of Latino and Asian populations (40% each) than of whites (20%) reside in the five states hit hardest by the housing recession: Arizona, California, Florida, Michigan, and Nevada (65). Yet, most of the wealth of REMs, unlike that of whites, stems from home ownership (Table 2). This means that REMs are experiencing ongoing decreases in wealth that exceed those of whites. Indeed, a larger percentage of REMs than of whites are underwater in their mortgages (owe more than their home is worth) in part because of where REMs’ homes are located (65), and in part because REMs pay significantly higher mortgage interest rates (45–51, 55). African-Americans are 86% more likely and Latinos 36% more likely than whites to be underwater in their mortgages (65). Thus, for REMs, owning a home often leads to poverty instead of to financial security (45–51, 55, 65–67).

**Table 2:** **Racial–ethnic differences in wealth (65)**.

	Whites	Blacks	Latinos	Asians
Home Ownership, 2011	98%	42%	43%	59%
2005–2010 Median Home Equity Decrease	−32%	−36%	−46%	−56%
% of Wealth based on Home Ownership	58%	92%	67%	72%
Loss of Wealth/Decrease in Net Worth, 2011	21%	45%	58%	48%
Median Liquid Wealth, 2011	$23,000	$200	$340	$19,500
Have Checking Accounts	80%	55%	60%	83%
Have Retirement Accounts	58%	32%	28%	57%
Have Other Assets (stocks, bonds, etc.)	31%	9%	6%	24%
% With Unsecured Debt, 2011	47%	44%	42%	45%

Home ownership is just one aspect of wealth. Other assets such as checking, savings, and retirement accounts also contribute. REMs are significantly less likely than whites to possess such assets and have significantly lower liquid wealth. Liquid wealth refers to assets that quickly can be converted into cash (liquidated). As shown in Table 2, the median liquid wealth of whites is 100 times that of African-Americans and 65 times that of Latinos. Liquid wealth “is largely non-existent within Black and Latino households,” [(65), p. 3]. If faced with a crisis in which cash assets must be used (e.g., checking and savings accounts) and other assets (e.g., retirement accounts, cars) sold for cash, Latinos and African-Americans have little. When subtracting retirement account dollars from the numbers shown in Table 2, African-Americans have $25, Latinos $100, and Whites $3,000 in liquid wealth (65). Two-thirds (67%) of African-Americans and 71% of Latinos (vs. 34% of Whites) are liquid asset poor, i.e., liquidating their assets is not sufficient to survive a crisis such as a death in the family or an accident or illness requiring expensive treatment, hospitalization, or long-term care (65–67).

Why do REMs have lower wealth and liquid wealth than whites? Do these differences reflect irresponsible financial behavior on the part of REMs, such as accumulating large unsecured debts (school loans, credit card, and medical bills) that render savings and other investment accounts difficult to build? As shown in Table 2, there are no racial–ethnic differences in unsecured debt (65). Instead, racial–ethnic differences in wealth reflect REMs’ lower incomes, combined with REMs paying more for housing, goods, and services with those lower incomes, and with the absence of banks in REM neighborhoods in which to establish checking and savings accounts (68). Like major department stores and chain-supermarkets (69, 70), banks began their flight from REM neighborhoods in the 1950s, and were replaced by predatory payday lenders and check-cashing houses (68).

Thus, unlike income, wealth captures the experience of SES in the US, i.e., being financially secure vs. insecure and vulnerable to minor (increased utility bills) or major (illness, death) crises (37, 65). Racial–ethnic differences in wealth hold across income (65), and wealth is a reliable measure that does not fluctuate monthly (65). Wealth is also a stronger predictor than income of health for all racial–ethnic groups (whites included), and wealth contributes to health independent of income (24, 37, 38, 67). In addition, REMs might exhibit lower non-response rates to questions about wealth (savings accounts, home ownership) than to questions about income. Moreover, wealth is an SES measure that is equivalent across racial–ethnic groups because it reflects rather than ignores the role of racial–ethnic discrimination in SES. Consequently, wealth is regarded as superior to income as a measure of SES in health disparities research (24, 37–39, 65, 67). Many have argued that the failure to measure wealth underestimates racial–ethnic differences in SES, and thereby underestimates the contribution of SES to racial–ethnic health disparities (24, 37, 38, 67).

##### Area measures of SES

The SES of a geographic area is another alternative to income. Area-SES can be assessed at any area level, i.e., census tracts (CT), zip-codes, counties, and states. Area-SES measures at the CT level are more robust than those at larger (zip-code, county) levels for examining racial–ethnic and other health disparities (71–75). The relationship between area-SES and health tends to be strongest when small areas (CTS) are used because larger areas contain small areas within them that vary considerably in area-SES and thereby reduce area-SES effects (71–75).

Irrespective of area level used, area-SES can be measured in a variety of ways. These include area median household income, area median home values, and composite measures such as the Townsend Index (71–75). When comparing 18 area-SES measures on their ability to predict health disparities, Krieger and her colleagues found that the percentage of CT residents below the federal poverty line (% BPL) is superior to other measures (71–74). For example, after controlling for household income, CT% BPL remains a strong predictor of racial–ethnic disparities in tuberculosis (73), smoking (76, 77), reproductive health and birth outcomes (78, 79), breast and cervical cancer screening (80), hypertension (81), cancer incidence, mortality and survival (82), and other health outcomes (73, 83). Poor health and health behaviors are significantly more prevalent in poor areas, i.e., where CT%BPL ≥ 20% (a federal poverty area) than in higher SES areas (CT%BPL ≤ 5% or 10%).

In general, SES measured at the CT level is a stronger predictor of racial–ethnic and other health disparities than SES measured at the household level (71–75). This is because area-SES captures the differences between low- and higher-SES areas in the hazards and resources that are known social-determinants of health (71–75). For example, compared to higher-SES areas, low-SES areas have significantly more prevalent hazards (84–100) including higher exposures to environmental toxins (84–89); poorer housing quality and higher indoor exposures to lead paint, carbon monoxide and other hazards (90–95); higher access to fast-food (96, 97); and greater prevalence of negative physical conditions such as garbage-filled vacant lots, wild dogs, abandoned buildings, and the absence of trees and sidewalks (98–100). In terms of resources, low-SES areas have significantly fewer grocery stores selling fresh fruit and vegetables (101–104) and fewer recreational facilities (101, 105–108), and lower availability and quality of healthcare (109–112).

These area-SES differences contribute to the higher prevalence of hypertension, diabetes, obesity, and other health problems (noted above) in low-SES areas (71–83, 90, 91, 95). Consequently, when people move out of low-SES areas into higher-SES areas, their health significantly improves even though their incomes remain unchanged. Evidence for this stems from the Moving to Opportunity (MTO) Experiment (113–116) and the many studies based on it (117–122).

##### Moving to opportunity (MTO)

The U.S. Department of Housing and Urban Development (HUD) conducted the MTO study between 1994 and 1998 in five cities: Baltimore, Boston, Chicago, Los Angeles, and New York. The study involved 4,604 low-income urban households (4,499 women and their 6,300 children) who resided in public-housing in extremely high poverty (CT%BPL ≥ 40%), high-segregated areas. The majority (93–96%) of the households were African-American (51–54%) and Latino (39–45%); all household incomes were below the federal poverty line; most (51–75%) households were receiving public assistance (AFDC, TANF); and few (22–30%) were employed. In the MTO study, a random half of these households (MTO group) received housing vouchers that could be used only to reside in higher-SES neighborhoods (≤10% CT% BPL), and half (non-MTO) remained in their poverty settings [see Ref. (113, 114) for details of the study design]. Measures of physical and mental health were taken at enrollment and at all follow-up years, and included height, weight (and calculation of BMI), glycated hemoglobin (HBA1c) tests for diabetes, and assessment of substance abuse and psychiatric disorders (via interviews using DSM diagnostic procedures and categories). The MTO and non-MTO groups were equal on all measures at baseline.

HUD’s 2001 (113, 114), 2003 (115), and 2012 follow-up analyses (116), as well as analyses conducted by others at 2-, 3-, 5-, 7-, and 10–15-year follow-up (115–122) all revealed the same results: Those who moved out of high-poverty areas (MTO group) exhibited significantly better physical and mental health than their counterparts who did not move, and exhibited positive changes over baseline (114–122). For example, significant decreases in the prevalence of morbid- and severe-obesity and in HbA1c were found among the MTO group (120, 122). The MTO study demonstrates that area income (rather than household income) predicts health among REMs. This is evident because the incomes of the REMs who moved did not change. Indeed, in the course of the study, the incomes, education levels, and employment of the REMs who relocated remained unchanged and similar to those of the non-MTO group (123, 124). Moving to a higher-SES area had positive effects on REM health, but had no effect on power, privilege or prestige because area-residence does not alter rigid status hierarchies based on race–ethnicity or SES, nor end racial–ethnic and SES discrimination (123, 124).

##### The area-SES of REMs

Area-SES and the MTO experiment are relevant because significantly greater percentages of REMs than of whites reside in low-SES areas (125). One in every four African-Americans, 1 in every 6 Latinos, and 1 in every 8 American Indians (vs. 1 in 25 whites) resides in a high-poverty area (CT%BPL ≥ 30%). Indeed, as a result of housing discrimination, many higher income REMs also reside in low-SES areas while their white counterparts with similar incomes do not (41, 45, 47, 51, 55, 125). Moreover, REMs are more likely than whites to reside in low-SES areas for all or most of their lifetimes, and thus are more likely to be subject to low-SES area health hazards and lack of health resources throughout the life course (58–63).

Because residence in poor areas predicts health better than income in general (71–75) and among African-Americans and Latinos in particular (MTO study), it is essential to measure area-SES in racial–ethnic health disparities research (71–75). Studies that fail to do so profoundly underestimate racial–ethnic differences in SES, and thereby underestimate the contribution of SES to racial–ethnic health disparities (71–75). Area-SES measures are also more valid and reliable than income because they are not subject to non-response bias, household size, or monthly fluctuations and the problem of cross-sectional SES measures. Moreover, area measures are equivalent across racial–ethnic groups because they reflect rather than ignore the racial–ethnic discrimination that relegates REMs to low-SES areas based on race–ethnicity and often irrespective of their incomes. Area-SES data (i.e., CT% BPL for every CT in the US) are publically and readily available to be included in studies of racial–ethnic health disparities (at www.hsph.harvard.edu/thegeocodingproject) (71–75).

#### Suggestions for measuring SES

Racial–ethnic groups differ on a variety of demographic variables that must be (but often are not) controlled in studies of health disparities (e.g., age, gender, and marital status, see our calculations in Table 3). Of all demographic correlates of race–ethnicity, SES is the most important to control. To do so, adequate measures of SES that are equivalent across racial–ethnic groups must be used. As shown here, wealth and area-SES are two such measures. Hence, we recommend using wealth and area-SES, along with household income, education, and household size to control for SES in studies of racial–ethnic health disparities, and improve the equivalence (comparability) of racial–ethnic samples. To our knowledge, no epidemiologic study has used all five measures. We also encourage those who found racial–ethnic health disparities to re-analyze their data using these five SES measures and examine the variability in health remaining after so doing. Use of all five measures does not guarantee that all variance in health due to SES will be accounted for, however, because other SES variables that correlate with race–ethnicity (e.g., wage theft, hazardous-working conditions, health insurance, and landline telephones) have not been controlled. It is beneficial to state this as a limitation of the recommended five measures, and to use tentative language in conclusions about racial–ethnic vs. SES health disparities.

**Table 3:** **Racial–ethnic differences in demographics among adults in the 2000 BRFSS**.

	Whites	African-Americans	Latinos	American Indians
	*N* = 85,543	*N* = 11,308	*N* = 9,080	*N* = 1,211
**Mean age**	47.36	42.77	38.89	42.35
Difference from Whites		−4.59 years*^a^	−8.47 years*^b^	−5.01 years*^c^
**Marital status**				
% Married, %Unmarried	76.5, 23.5	48.5, 51.5*^d^	67.5, 32.5*^e^	65.3, 34.7*^f^
**Gender**
% Men, % Women	40.9, 59.1	34.0, 66.0*^g^	41.1, 58.9^NS^	42.6, 57.4^NS^

Comparison to Whites; ^a^*t* = 27.077; ^b^*t* = 45.45; ^c^*t* = 10.11; ^d^χ*^2^* = 2617.02; ^e^χ*^2^* = 263.79; ^f^χ*^2^* = 57.47; ^g^χ*^2^* = 197.59; *p < 0.0005; NS = not significant.

#### Measuring and controlling segregation to improve sample equivalence

All racial–ethnic groups reside in areas that differ in area-SES and in racial–ethnic segregation as well. Segregation contributes significantly to the health of whites and of all REMs (126–129), but most research has focused on blacks. Thus, many examples here focus on black–white segregation.

Residential segregation refers to the geographic separation of whites from REMs in residential areas (130–132). Like area-SES, segregation can be measured at any area level such as CTS, states, and metropolitan statistical areas (MSAs). Measuring segregation in smaller (CTS) areas is preferred because large areas (e.g., counties) contain both highly segregated and integrated smaller areas within them. Measuring segregation at the CT level, or at the MSA level (with MSA-segregation calculated from CT data) is widely regarded as superior and is generally preferred (130–132). Irrespective of area level used, however, segregation can be measured in many ways (Table 4), including dissimilarity, isolation, concentration, clustering, centralization, and hypersegregation (133–139). Of these, dissimilarity is most often used, but Isolation has better validity and interpretability (134, 138, 139). Crude measures of questionable validity (e.g., area-percentage of blacks) often are used as well. The segregation–health relationship varies with the segregation measure used (133–139).

**Table 4:** **Dimensions and measures of residential segregation^a^ (133)**.

Dimension/measure	Definition
Dissimilarity	The distribution of whites vs. a minority group across residential areas, resulting in mostly white vs. mostly minority neighborhoods. Interpreted as the percentage of the minority group who would have to move to achieve residential integration. Referred to as the *Segregation Index* (SI), and as the *Dissimilarity Index* (DM).
Isolation/exposure	The average probability of contact between minority group members and whites in residential neighborhoods. Referred to as the *Isolation Index*.
Concentration	The population density of segregated minority areas; the amount of physical space occupied by the segregated minority group vs. Whites.
Clustering	The degree to which minority neighborhoods are adjacent to each other vs. dispersed; high clustering refers to several adjacent minority neighborhoods that constitute enclaves, ghettos, niches, or barrios.
Centralization	The degree to which minority neighborhoods are located near a metropolitan area’s urban center (vs. its suburbs).
Hypersegregation	The simultaneous occurrence of all of the above.

*^a^Each measure is calculated separately for each minority vs. Whites, i.e., Black-White SI, Latino-White SI*.

Dissimilarity (SI) data are calculated by the U.S. Census Bureau and are available in census datasets. SI ranges from 0 (a fully integrated city in which blacks and whites reside in all areas) to 100 (a totally segregated city in which all blacks and whites reside in separate neighborhoods). Hence, SI is interpreted as the percentage of blacks who would have to move to achieve city-wide integration. SI data indicate that the US continues to be characterized by high black–white segregation: Nationwide, 60-70% (most) of blacks and 70–90% of whites reside in mostly black and mostly white areas (respectively), meaning that 60–70% of blacks would have to move to integrate most US cities.

For example, as shown in Table 5 (2010 column for Black–White SI), 81.5% of the blacks in Milwaukee would have to move to integrate that city, as would 75.3% of blacks in Detroit, and 76.4% of blacks in Chicago. A high (>60) or very high (>70) SI means that irrespective of their representation in a city’s population, most blacks and whites reside in more or less racially homogenous areas where they are isolated from and rarely exposed to each other. For example, as shown by the Isolation – Exposure data in Table 6 blacks comprise about 23% of Detroit’s population, but live in neighborhoods that are 80% black. Likewise, blacks constitute about 18% of Chicago’s population, but live in neighborhoods that are 75% black. If Chicago were an integrated city, every neighborhood would consist of 58% whites, 18.6% blacks, and 17.1% Latinos in manner matching their representation in that city’s population (Table 6); in the absence of racial segregation, Chicago blacks would reside in neighborhoods that are 18.6% rather than 75% black.

**Table 5:** **Segregation Index (SI) data for 10 metropolitan statistical areas (MSAs): 1990, 2000, and 2010 US Census**.

Metropolitan statistical area (MSA)	Black-White SI			Latino-White SI			Asian-White SI		
	1990	2000	2010	1990	2000	2010	1990	2000	2010
Baltimore	71.4	68.2	65.4	30.2	35.8	39.8	38.3	41.1	43.6
Chicago	84.4	81.2	76.4	61.4	60.7	56.3	46.5	46.8	44.9
Cleveland	82.8	78.2	74.1	58.3	58.5	52.3	38.1	39.9	41.3
Detroit, MI	87.6	85.7	75.3	40.2	46.0	43.3	43.1	48.8	50.6
Los Angeles	72.7	70.0	67.8	60.3	62.5	62.2	43.5	47.9	48.4
Miami, FL	71.4	69.2	64.8	32.5	59.0	57.4	26.8	33.3	34.2
Milwaukee	82.8	83.3	81.5	56.4	59.5	57.0	42.2	43.4	40.7
New York	80.9	80.2	78.0	66.2	65.6	62.0	47.4	50.8	51.9
Philadelphia	75.2	71.0	68.4	60.9	58.5	55.1	42.4	44.1	42.3
St. Louis	77.2	74.1	72.3	23.5	27.7	30.7	39.8	45.2	44.3

*Source: http://www.censusscope.org*.

**Table 6:** **Exposure/isolation in three major US cities, 2000**.

			Composition of neighborhoods of residence		
% of City’s population			% White	% Black	% Latino
Detroit	Whites	69.7%	87.9	4.9	2.5
	Blacks	22.8%	15.0	80.0	1.7
	Latinos	2.9%	61.4	13.6	20.1
Chicago	Whites	58.0%	78.6	4.5	10.5
	Blacks	18.6%	14.0	75.4	7.5
	Latinos	17.1%	35.6	8.2	50.7
Atlanta	Whites	59.8%	77.9	12.5	4.9
	Blacks	28.7%	26.0	64.4	5.6
	Latinos	6.5%	45.2	24.4	23.2

*Data Source: http://www.censusscope.org*.

These segregation levels have been somewhat stable for all REMs for decades (Table 5), with the exception of increases in the segregation of Latinos (1990–2000) and Asians (1990–2010) in large cities (25, 127, 131, 140), and declines in the segregation of blacks (125, 127). The decreases in black segregation, however, have been small each decade, and black segregation nonetheless remains high or very high, i.e., SI > 60 or 70 [Table 5; (125, 127, 130, 131)]. Likewise, although all REMs are segregated to some extent, the segregation levels of blacks continue to be significantly higher than those of all other REMs (Table 5). Likewise, blacks are more racially isolated than other REMs. For example, as shown in Table 6, blacks and Latinos both constitute about 18% of Chicago’s population, but blacks live in neighborhoods that are 75% black and 14% white, whereas Latinos live in (more integrated) areas that are 50% Latino and 36% white. Moreover, although all REMs are segregated along one or more dimensions of segregation (Table 4), blacks are more likely than others to be hypersegregated, i.e., along all 5 dimensions (137): Blacks are more likely than other REMs to live in densely populated (Concentration), mostly black (Dissimilarity) neighborhoods that are adjacent to similar neighborhoods (Clustering), in the center of cities (Centralization), and isolated from whites (Exposure/Isolation). About 60–70% of the black population resides in segregated black areas, and 40–50% reside in hypersegregated black areas (137, 141).

This “American Apartheid” (132) generally does not reflect a black preference to live in black neighborhoods (142, 143), but instead is primarily the result of the discriminatory housing practices noted previously here (45–51). In large surveys, most blacks indicate a preference to live (and raise their children) in integrated neighborhoods, but Whites do not want them (142–145) and threaten to move out (of affluent areas in particular) if blacks move in (142–145). Real estate agents actualize these white preferences by steering black home-buyers and renters away from mostly white areas via the false message that the property is no longer available and the failure to show the property to blacks (45–51). The discriminatory housing practices that relegate most blacks to mostly black neighborhoods hold across black household income, and most strongly affect high income blacks who attempt to purchase homes in affluent, white suburbs (146–148). High income blacks often are as segregated as their low income cohorts but reside in affluent rather than in poor black neighborhoods (146–148). Thus, residential racial segregation does not mirror residential income segregation and is not an artifact of black–white differences in income (142–149). Indeed, if the US population was distributed into residential areas based solely on income, the US would be very integrated (144, 149). For example, based on income (not race), the SI for St. Louis, MO would be 11 rather than 78 (149).

##### Segregation and black health

Numerous studies have found that the health of blacks who reside in high-segregated black neighborhoods is significantly worse than that of their less-segregated counterparts. Compared to blacks who reside in low-segregated areas, high-segregated blacks have significantly higher: adult mortality (150, 151) and infectious diseases rates (152); prevalence of adverse birth outcomes such as low birth-weight and preterm birth (27, 141, 153–155); prevalence of obesity (138, 156–159); cardiovascular disease mortality (160, 161); prevalence of asthma (162, 163) and hypertension (164, 165); breast and lung cancer mortality (166, 167); cancer risks due to exposure to air toxics (168, 169); prevalence of poor self-rated health (170) and of drug use (171); lower rates of physical activity (172) and of fruit/vegetable consumption (157), and other health problems (126–129, 134, 173). Most of the health problems on which black–white disparities have been found are significantly more prevalent among high- than among low-segregated blacks.

The poorer health of high-segregated African-Americans is generally understood as a function of the significantly higher exposures to health hazards and the significantly lower availability of health-enhancing resources in high-segregated black neighborhoods (28, 29, 126–128). High-segregated black areas, compared to low-segregated ones, are characterized by higher exposure to air toxics and persistent organic pollutants (173, 174); few grocery stores selling fresh fruit/vegetables and a higher prevalence of food deserts (175–178); lower availability of recreational facilities (106, 107); poorer quality housing (92–95, 179) and neighborhood physical conditions (98–100, 180); higher prevalence and density of fast-food places (96, 97, 181, 182); and low access to specialized and to high-quality physicians and healthcare facilities (128, 183–186).

##### Effects of black segregation on whites’ health

Residing in a segregated black neighborhood is associated with poor health and health behavior among all residents irrespective of their race–ethnicity [e.g., Ref. (129, 172, 187)]. For example, residing in a segregated black neighborhood is associated with increased physical inactivity (172), and with lower cancer screening (11, 129) among blacks and whites alike. Similarly, in a recent study, we found that black cancer survivors had significantly poorer health-related quality of life than white survivors (187) even after controlling for income and area-SES (%BPL). When segregation (black Isolation Index) was added to the regressions, however, racial differences disappeared; health-related quality of life was poor among all cancer survivors who resided in mostly black areas. Such findings highlight that the black-segregation effect is not a function of the people but of the place, i.e., of high health hazards and low health resources that affect all residents.

##### Segregation and health among other REMs

**Figure 1:**
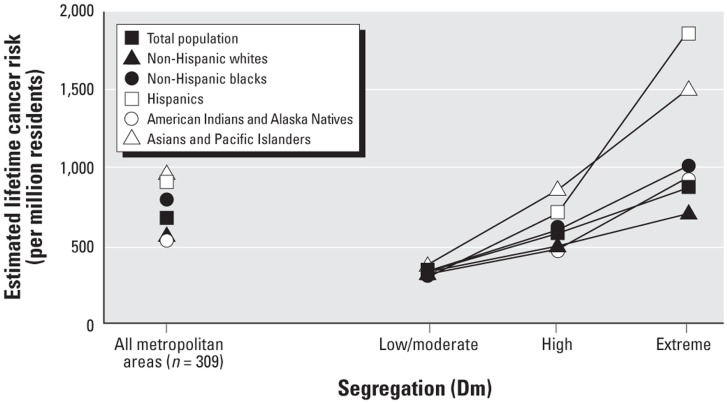
**Estimated lifetime cancer risk associated with exposure to ambient air toxics in low, high, and extremely-high segregated neighborhoods**.

There are relatively few studies of segregation and health among other REMs. The growing literature on Latinos, however, reveals that many findings for segregated blacks hold for segregated Latinos as well. For example, Latinos who reside in high-segregated Latino neighborhoods (compared to those who reside in low-segregated areas) have significantly higher prevalence of preterm birth (155) and of obesity (158, 188), and lower levels of physical activity (189). Moreover, the cancer risk associated with exposure to air toxics is higher in black, Latino, Native American, and Asian neighborhoods than in white neighborhoods (29). Indeed, such exposure and risk are highest in extremely segregated Latino neighborhoods and lowest in extremely segregated white neighborhood as shown in Figure 1 [(29), p. 391].

Although many of the negative health outcomes associated with high segregation for blacks hold for high-segregated Latinos (29, 155, 158, 188), some studies have found that residing in a segregated Latino area has no or has positive effects on Latino health (190, 191). This is in part due to blacks’ higher segregation than that of Latinos ((140, 192, 193); Tables 5 and 6), and the significant differences between segregated Latino areas in nativity (percent foreign-born), Latino ethnic group (e.g., Mexican vs. Puerto Rican), and language spoken (190, 191). Nonetheless, however, studies of segregation and health among other REMs have revealed that the resource-poor and hazard-rich conditions found in high-segregated black areas characterize high-segregated Latino and Asian neighborhoods as well (180, 185, 186, 193), and contribute to the health disparities of Latinos and Asians (173, 194).

#### Suggestions for including segregation

Data strongly suggest that it is useful to measure and control for the segregation of REMs and of whites in research on racial–ethnic health disparities (29, 30, 140, 155, 158, 188–194). Doing so controls for the health-relevant area hazards and built environments experienced by REMs vs. whites (29, 140, 167–174, 180–193, 195, 196), and thereby improves racial–ethnic sample equivalence. Dissimilarity or isolation at the CT or MSA level is the common strategy for including segregation in health disparities research [e.g., Ref. (29, 187)]. However, controlling for segregation does not guarantee that all racial–ethnic differences in place (e.g., urban vs. suburban residence) are controlled. Thus, it is beneficial to state this as a limitation of controlling for segregation, and to use tentative language in conclusions about racial–ethnic vs. place-related health disparities.

## Method Equivalence

Method equivalence is the extent to which the methodology used to acquire health data is equal for the status groups in the research; method equivalence exists when all status groups are equally familiar with the methods and have equal opportunity to participate in the study (197–201). Here, we focus on only two of the types of method equivalence.

### Familiarity-related method equivalence

Familiarity-related method equivalence exists when all status groups are equally familiar with research methods such as reading, writing, taking tests, being timed, being interviewed (i.e., one-sided conversations), answering multiple-choice and Likert-type items, etc. (198–201). Racial–ethnic (and SES) groups always differ in their familiarity with at least one of these methods because they differ in their frequency of engaging in these activities outside of the study (198–201). Thus, some degree of familiarity-related method bias is present in all research with diverse samples, and is evident in participants’ questions about the tasks as well as in the tendency of REMS to ask interviewers how they would answer the interview questions (198–201).

#### Improving equivalence

An effective way to test and control for lack of familiarity-related method equivalence is test–retest, i.e., the repeated administration of the task. On retest, all groups are likely to exhibit slight increases in performance or more consistent responses as a function of practice effects. However, when lack of method equivalence across racial–ethnic groups exists, the changes in responses for REMs significantly exceed those for Whites, and indicate that prior REM responses were in part an artifact of lack of familiarity with the methods (198–201). Statistically significant racial–ethnic differences in test–retest reliability coefficients are used to assess familiarity-related method equivalence (198–201), and hence we recommended its use with all (or a subset of) participants.

### Inclusion-related method equivalence: Language

Inclusion-related method equivalence exists when all groups have equal opportunity to participate in the study. Methodologies that differentially exclude REMs and low-SES groups from participation lack this type of method equivalence. Two such normative methods are English-only studies, and random digit-dial telephone surveys (RDDTS).

#### English-only studies

More than 80% of the studies in medical and public health journals were conducted in English-only, with non-English speakers excluded (202–204). The vast majority of epidemiologic studies of health disparities, ranging from small studies to state- and nationwide population-health surveys (e.g., Current Population Survey, BRFSS) are conducted in English, or at best in English and Spanish (202–204). The exclusion of non-English speakers decreases racial–ethnic sample equivalence and the validity of data from REMs, and also yields non-representative REM samples from whom generalization to their populations is severely limited (202–204).

Specifically, English-fluent samples exclude the approximately 40% of Asians and of Latinos who do not speak English well (202–204). Because English proficiency is a proxy for nativity, years of residence in the US, education, and income (205, 206), the 40% of Asians and Latinos excluded tend to be the recent-immigrant, low-SES, and low-educated members of these groups (203). English-fluent samples thereby provide estimates of Asian and Latino SES that are significantly higher than those of their populations, and hence underestimate the contribution of SES to health disparities among Latinos and Asians (202–204). Moreover, lack of fluency in English is a known barrier to health care (203). Non-English speaking Latinos and Asians are significantly less likely than their English-fluent cohorts to have had immunizations, cancer-screening tests, preventive services, or even adequate physical and mental health treatments, irrespective of their SES and health insurance (109, 186, 206–208). Studies of English-fluent Asians and Latinos thus provide not only grossly inaccurate demographic data but also erroneous estimates of the health of these groups as well. Data from the 2003 California Health Interview Survey (CHIS) illustrate these points.

The CHIS was a statewide, RDDTS of the physical and mental health and health behaviors of California adults (www.CHIS.ucla.edu). Unlike other population-health surveys, the CHIS was conducted in English, Spanish, Chinese (Mandarin and Cantonese), Korean, and Vietnamese. Table 7 compares Asians and Latinos who participated in the CHIS in English (English-fluent) vs. another language (non-fluent). As shown, Asians and Latinos interviewed in English had incomes (mean $70,500) nearly three times higher than those of their cohorts interviewed in other languages (mean $24,700), a difference of $45,800 annually. Likewise, English-fluent Asians and Latinos were significantly more educated than those non-fluent, and had spent a significantly greater percentage of their lives in the US. English-fluent Asians also were significantly younger (by as much as 15 years) than their non-fluent cohorts, and the English-fluent Asian samples contained significantly lower percentages of women (i.e., the differential exclusion of REM women).

**Table 7:** **Differences between English-fluent and non-fluent Latinos and Asians in the 2003 California Health Interview Survey (CHIS)^a^^,^^b^**.

CHIS language		Mean age	% Women	% of Lifetime spent in USA	% ≤High school education	Mean income	% Fair/poor self-rated health	% Have health insurance	% With diagnosed hypertension
Latinos	English	37.5	50.7	89.9	53.9	$51,900	17.6	81.0	19.3
	Spanish	39.0	48.0*	38.8*	90.8*	$21,800*	44.4*	55.0*	16.7*
Asians (all)	English	41.0	50.5	27.3	21.0	$76,300	12.5	90.9	20.7
	Other	49.9*	58.9*	17.9*	56.5*	$38,800*	44.0*	76.7*	25.9*
Chinese	English	40.0	51.8	65.2	15.4	$82,100	5.9	92.7	13.0
	Chinese	52.2*	61.0*	28.2*	59.3*	$34,000*	41.0*	76.1*	26.7*
Koreans	English	34.8	47.5	67.9	25.8	$90,800	7.5	76.0	14.3
	Korean	48.1*	64.8*	30.5*	34.3*	$61,300*	35.5*	66.8*	15.6
Vietnamese	English	32.0	38.8	65.8	21.0	$82,200	10.6	86.5	10.8
	Vietnamese	47.2*	53.3*	28.4*	68.1*	$29,300*	56.9*	80.9*	28.9*

*^a^Data culled from multiple tables in Ref. (203)*.

*^b^*N* = 42,044 Latinos and Asians*.

*p < 0.001 for each language comparison.

Where health is concerned, a significantly greater percentage of English-fluent Asians and Latinos had health insurance, and significantly fewer rated their health as fair/poor. For example, a mere 5.9% of English speaking vs. 41% of Chinese-speaking Chinese rated their health as fair/poor, and 17.6% of English speaking vs. 44.4% of Spanish-speaking Latinos rated their health as fair/poor. Non-English speakers also often had a significantly higher prevalence of hypertension (Table 7); they often had higher rates of diabetes, had never seen a dentist, and reported greater discrimination in health care as well (data not shown). Such findings highlight that English-speaking Asian and Latino samples do not represent their populations’ demographics or health, underestimate Asian and Latino health disparities, and underestimate the contribution of SES to those disparities (203, 204, 206–209).

#### Reducing language-related method bias

Non-participation in research because it is conducted in English-only requires correction to increase the validity of data on Asians and Latinos, and improve the representativeness of such samples (203, 209). The obvious solution is to translate epidemiologic surveys into the non-English languages spoken most often in the US, i.e., Spanish and Chinese, and perhaps Vietnamese and Korean as well. Although such translations increase the cost of the research, cost is not an acceptable reason for excluding REMs and their sub-populations (those not fluent in English) according to NIH guidelines for inclusion of women and minorities in research (210). Those guidelines however refer to clinical trials rather than to epidemiologic studies of health disparities. Hence, reducing language-related method bias (increasing method equivalence for all REMs) inevitably may require revision of the NIH guidelines to address language-related exclusion in all NIH-supported research. We strongly recommend such revision.

### Inclusion-related method equivalence: RDDTS

The RDDTS method involves telephoning randomly selected household landline telephone numbers and administering a standardized health survey. The method has been used in population-health surveys (e.g., BRFSS) since the 1980s and 1990s to acquire random, representative samples, and hence RDDTS data provide much of the evidence on population racial–ethnic health disparities.

#### 20th century non-coverage bias

Since the 1980s and 1990s, however, data from NHIS and NHANES samples (i.e., people interviewed in person at home) revealed that REMs and low-SES groups were significantly more likely than others to lack landline phones (to be phoneless), and hence were being differentially excluded from RDDTS (211–215). For example, Anderson et al. (215) analyzed 1991–1994 NHIS data and found that 5% were phoneless: 10% of blacks and Latinos (vs. 3% of Whites) interviewed at home, 17% of those at and below the poverty line, and 21% of blacks at and below the poverty line were phoneless. The Census Bureau (216) similarly found that 5% of the 1990 population was phoneless, with American Indians (23%), blacks (13%), and Latinos (12%) most likely to lack phones. The data also indicated that minority RDDTS samples from the 1990s had higher SES levels than their phoneless cohorts, and hence were not representative of their populations (211–215).

#### 21st century non-coverage bias

In the 2000s, the prevalence of non-coverage (lack of landlines) increased for the US population as people began substituting less expensive cellular phones for landlines (217–220). The percentage of US wireless (cell phone only) households was 7.2% in 2004, 8.4% in 2005, 12.8% in 2006 (217–220), and 40.6% in 2012 (221). Data in the 2000s reveal that wireless households are significantly more likely to live in poverty and to be REMs than landline households, and than households with landlines and cell phones (217–220). Demographic data on wireless US households in 2012 are shown in Table 8. Moreover, health among wireless and phoneless households continues to be significantly poorer than that of landline households. For example, Blumberg et al. (218) compared 2004–2005 NHIS participants who had vs. lacked landlines. Those without landlines were significantly more likely to lack health insurance (e.g., landline = 14.8%, wireless only = 31.1%, phoneless = 43.9%), engage in binge drinking, report serious psychological distress, and be current smokers (landline = 19.7%, wireless = 32.9%, phoneless = 36.9%). Likewise, 2012 data (221) revealed that wireless only households continue to have a higher prevalence of binge drinking (wireless = 30.5%, landline = 17.5%), cigarette smoking (wireless = 24.3%, landline = 17.5%), lack of health insurance (wireless = 27.9%, landline = 15.1%), and failure to obtain health care because of financial barriers (wireless = 12.2%, landline = 6.0%).

**Table 8:** **Demographics of wireless only households, 2012^a^^,^^b^**.

Ethnicity		Age		Poverty and property	
Whites	30.4	18–24	49.5	Poor	51.8
Latinos	46.5	25–29	60.1	Near Poor	42.3
African-Americans	37.7	30–34	55.1	Not Poor	30.7
Asians	33.4	35–44	39.1		
Other Ethnic Minorities	43.4	45–64	25.8	Home owner	23.2
Multi-racial	40.2	≥65	10.5	Renting	58.2

*^a^Data culled from multiple tables in Ref. (221)*.

*^b^*N* = 13,724*.

Such data highlight the non-equivalence of the RDDTS method by race–ethnicity and SES, i.e., the differential exclusion of REMs and low-SES groups due to their lack of landlines. As noted, the result of such exclusion is that REM RDDTS samples have significantly higher SES levels and better health than their wireless counterparts who cannot participate in RDDTS (211–221). This has led to questions about the validity and the population-representativeness of demographic and health data from REM RDDTS samples. Of greatest concern is that REM RDDTS samples underestimate the contribution of SES to racial–ethnic health disparities by excluding the poorest and least educated REMs. For example, in a recent study (222), we compared the demographics and cigarette smoking of a random, statewide, California (CA) sample of black adults surveyed door to door in person (*N* = 2218), to those of a random, statewide, CA RDDTS (i.e., CHIS) sample of blacks (*N* = 2315) acquired simultaneously. Results revealed that the in-person black sample was significantly younger, poorer, and less-educated than the RDDTS sample, and had significantly higher smoking prevalence, 32.6% (in-person sample) vs. 19.1% (RDDTS sample), even when controlling for demographics. Moreover, 13% of the in-person black sample was phoneless/wireless only, and hence could not participate in the RDDTS study. The in-person, phoneless/wireless group of blacks was the youngest, poorest, and least educated of all groups, and had the highest smoking prevalence – 50.2% overall, 47.7% for wireless/phoneless black men, and 53.4% for wireless/phoneless black women.

Other studies have revealed that REMs are more likely than whites to refuse to participate in RDDTS (with response rates ranging from 0.2 to 10%), and that REMs who reside in segregated areas are less likely to be phoned and less likely to participate if telephoned (202, 222, 223).

#### Reducing RDDTS exclusion-related method bias

Increasing concerns regarding non-coverage bias led the Centers for Disease Control (CDC) to begin to include wireless phone numbers in the BRFSS (224). Unfortunately, this change was not implemented until 2011. Moreover, the percentage of wireless phone numbers called remains too small to overcome the differential exclusion of REMs and of the low-SES. The median percentage of wireless numbers (of all phone numbers) called was 11% in the 2011 BRFSS, and 20% in the 2012 BRFSS (224). The RDDTS method thereby remains a biased one that acquires REM samples whose higher SES and superior health do not represent their populations, and the method continues to underestimate the role of SES in racial–ethnic health disparities. Hence, we urge epidemiologists who analyze and cite RDDTS data (e.g., 2000 BRFSS) as evidence of racial–ethnic health disparities to highlight the non-representativeness of the REM samples, and to draw extremely tentative conclusions about racial–ethnic health disparities – particularly given that the majority of RDDTS are conducted in English and Spanish only (i.e., exhibit language-related method bias as well). We also urge the CDC to include more wireless households in the BRFSS, and recommend that wireless households be over-sampled until the percentage that participates in the BRFSS is equal to the percentage of wireless households in the US.

## Scalar Equivalence

Scalar (scaling) equivalence refers to the extent to which the response categories provided for items (e.g., true/false, Likert-type scales) are responded to in the same way by all status groups, such that group differences found are responses to item content rather than item scaling (225–229).

### High vs. low-frequency scales and item order

Studies indicate that everyone’s responses to scaled items are partially a function of the response categories (the numbers and their labels) provided. For example, people assume that scale numbers and labels represent the researcher’s knowledge of the distribution of behaviors and symptoms, and assume that the midpoint of the scale is the norm. Hence, people report significantly higher frequencies of behaviors, symptoms, and events on scales with high-frequency (e.g., 1–10) than on scales with low-frequency (0–5) numbers (228, 229) because a higher-frequency is the norm (midpoint) on high-frequency scales. The high- vs. low-frequency scaling effect has been found in self-reports of the frequency of physical symptoms, psychiatric symptoms, health behaviors, and negative emotions, each higher on high-frequency scales (228, 229).

High- vs. low-frequency scaling also shapes self-perceptions and subsequent health-behavior intentions. For example, in one study, the number of sexual partners was presented on a low-frequency (0, 1, 2, 3 or more) and on a high-frequency (2, 3, 4, 5, 6, 7, 8, 9, 10 or more) scale. People who had 3 or more sexual partners rated their sexual behavior as risky and reported future intentions to use condoms when they received the low-frequency scale on which they understood 3 to be extreme and abnormal (230). Because scaling provides tacit information about norms, low-frequency scaling can be used unobtrusively to increase risk perceptions and healthy behavioral intentions (230). Scaling influences self-reports and self-perceptions because responding to quantitatively scaled items is a complex cognitive process involving interpreting each item’s content and its response categories; estimating one’s frequency of the behavior, symptom, or event in the past; using the response categories as a clue to norms; editing one’s response in a manner consistent with norms and with social desirability, and then finally answering the question (229–233). The order in which questions appear also influences responses to scaled items (234). As discussed later here, item order (context) influences REM but not white responses to the self-rated health item.

#### REM responses to scaled items

Scaling contributes to racial–ethnic differences on items, with REMs affected more strongly than whites (225, 226, 234–237). Likert-scales that range from 1 to 5, with labels that range from Strongly Disagree to Strongly Agree, or from Poor to Excellent, are often used in health research and are the most troublesome. This is because racial–ethnic differences in responses to scaling occur most often to such scales, and such scales are non-equivalent across racial–ethnic groups. Specifically, numerous studies have documented three, reliable ethnic-minority response styles to Likert-scales, the Acquiescent, Extreme, and Middle Response Styles (236–244). The Acquiescent Response Style is the tendency to agree/strongly agree with items irrespective of their content; the Extreme Response Style is the tendency to choose the extreme response (highest and lowest numbers) irrespective of item content; and the Middle Response Style is the tendency to select the midpoint of Likert-scales irrespective of item content (236–244). African-Americans and Latinos tend to exhibit Extreme, Asian Americans tend to exhibit Middle, and Latinos also often exhibit Acquiescent responses to five-point (in particular) and seven-point Likert-scales (236–244).

Many have suggested that the Extreme style reflects the REM-cultural value of providing clear, unambiguous responses; that the Acquiescent style reflects REM-cultural valuing of being polite, agreeable, and respectful; and that the Middle style reflects the REM-cultural desire to conform to norms and be similar to others (236–244). Thus, it has been hypothesized that all three response styles are manifestations of the universal cultural values and tendencies of Individualism–Collectivism, Uncertainty Avoidance, and Power Distance on which REMs generally tend to differ from whites (236–244). These terms are defined in Table 9.

**Table 9:** **Three dimensions of culture/cultural values (245–248)**.

**Individualism-Collectivism** is the extent to which the individual vs. the family has priority and primacy in a culture. Individualism is the extent to which a culture shapes, reinforces, and values independent adults and children who seek to stand-out from others, pursue their own goals and desires despite family views, move-away from parents’ home, and value and expect to have inviolable rights. Collectivism is the extent to which a culture shapes, reinforces and values interdependent adults and children who seek to fit-in harmoniously with others, pursue the family’s goals and desires despite their own, remain living with parents, and value and expect to have (not rights but) inviolable family duties and obligations.
**Power Distance** is the extent to which members of a culture accept existing status and power hierarchies (e.g., by age, gender, SES) as natural, normal, desirable, and permanent, and hence is the extent to which they value knowing and conforming to (high power distance) vs. ignoring and violating (low power distance) status-based interactional etiquette.
**Uncertainty Avoidance** is the extent to which members of a culture are uncomfortable with ambiguity and hence the steps they take to reduce it. This is manifested as the desire, need, and expectation (in high UA cultures) vs. the dislike and rejection (in low UA cultures) of specific and explicit rules, deadlines, guidance, supervision, goals, expectations, and evaluation criteria.
Each of the above is what everybody takes for granted as how people and things are and should be.

Individualism–Collectivism, Uncertainty Avoidance, and Power Distance tendencies are found among the members of all cultures in the US and worldwide, i.e., for example, every culture contains people who are Individualists or Collectivists. However, on the whole and for the most part, western-Europeans and European-Americans (US whites) tend to be high on Individualism and low on both Power Distance and Uncertainty Avoidance (Table 10). Hispanic and African people worldwide (Table 10), and Latinos and Native- and African-Americans in the US tend to be high on Collectivism, Power Distance, and Uncertainty Avoidance. Asians in the US and abroad (Table 10) tend to be high on Collectivism and high on Power Distance, but vary considerably (from low to very high) on Uncertainty Avoidance (239–248), as shown in the examples provided in Table 10.

**Table 10:** **Mean individualism, power distance, and uncertainty avoidance survey scores of 88,000 IBM employees in selected countries (245–247)**.

Individualism (very low to highest)		Power distance (very low to highest)		Uncertainty avoidance (very low to highest)	
6–8	Guatemala, Ecuador	11–18	Austria, Israel, Denmark	8–29	Singapore, Jamaica, Hong Kong, Sweden, Denmark
12–15	Venezuela, Indonesia, Pakistan, Costa Rica	28–34	Ireland, Sweden, Finland, Norway, Switzerland	35–45	England, India, Malaysia, Philippines
16–19	Peru, Taiwan, South Korea, El Salvador	35–39	England, Germany, Canada, Netherlands	**46**	**United States**
20–30	Thailand, West Africa, Chile, Hong Kong, East Africa, El Salvador South Korea, Malaysia, Mexico	**40**	**United States**	52–69	East Africa, Thailand Taiwan
38–51	Brazil, Jamaica, Argentina, Japan, India, Turkey	45–55	Argentina, Jamaica Pakistan, Japan	70–82	Pakistan, Brazil, Venezuela, Columbia, Mexico
80–90	England, Australia, Canada, Netherlands	63–77	Chile, Peru, Thailand, Hong Kong, Brazil	86–92	Costa Rica, Peru, Chile, Panama, Argentina
**91**	**United States**	81–100	Venezuela, Philippines, Malaysia, Guatemala, Panama, Mexico	>92	Japan, El Salvador, Guatemala, Uruguay

Researchers have theorized that the Acquiescent, Extreme, and (especially) the Middle Response Styles reflect high Collectivism, and that the Extreme style also reflects high uncertainty avoidance and high power distance (236–244). There is growing empirical support for the relationship between high Collectivism and the three response styles among US REMs and among people in Asian, African, and Latinos cultures worldwide (236–244). Data on the relationships between the response styles and power distance and uncertainty avoidance are less consistent (236–244, 249, 250). In addition, several studies have found that US low-SES, older (≥65 years), and low-educated populations also tend to exhibit the Acquiescent style on Likert-scales (234, 238, 241, 249, 250).

#### Response-style effects on REM health data

Irrespective of their sociocultural genesis and correlates, the Extreme, Acquiescent, and Middle Response Styles reveal that five-point Likert-scales are non-equivalent across racial–ethnic groups, and are a threat to the validity of health data from REMs (225, 226, 228, 238, 240–244). This is because REM response styles can be misinterpreted as substantive and yield false group differences. Likewise, statistical analyses are undermined by the response styles. The Extreme Style inflates standard deviations and decreases correlations, whereas the Acquiescent Style can yield a spurious factor composed of negatively keyed items, and result in failed confirmatory factor analyses with REMs (225, 226, 228, 238, 240–244). Epidemiologic studies of health disparities do not test or control for REM response styles, and thereby continue to raise the question of whether racial–ethnic disparities on the Likert-scaled items in health surveys are genuine differences or are simply artifacts of REM response styles (225, 226, 228, 238, 240–244).

#### Testing and controlling for response styles

Several strategies have been suggested to test and control for response styles. Foremost among these is changing the scaling used. Likert-scales that range from 1 to 5 (or 1 to 7) are most likely to elicit the response styles because they provide extreme anchors around a midpoint (225, 226, 235, 240, 249–253). Studies have demonstrated empirically that four- or six-point Likert-scales (i.e., without a midpoint) diminish all three response styles (235, 240, 249–253). Likewise, a mix of positive and negative-phrased items decreases the Acquiescent Style because people cannot simply agree with all items (225, 226, 228, 238, 240–244). In addition, several standardization methods and use of standardized instead of raw scores have been suggested (249, 251–255). Other suggestions include computing the proportion of items endorsed (agreed with) to measure Acquiescent responding, and the proportion of extreme responses to measure Extreme responding, administering the *Greenleaf Extreme Response Scale* along with the study measures (256, 257), and use of structural equation modeling and item-response theory (225, 226, 240–244, 249, 251–255).

#### The trouble with self-rated health

A question on self-rated health is included in almost all epidemiologic studies, such as the BRFSS, NHANES, NHIS, CHIS, and international population-health surveys (258–261). In general, self-rated health has a strong relationship to adult mortality (260, 261), although the strength of that relationship varies by SES (262), marital status (263), education (264), race–ethnicity and gender (265), and residential segregation (26, 170, 266). Nonetheless, self-rated health is widely regarded as a valid, valuable measure of population health and health trajectories, and hence often is used in studies of racial–ethnic health disparities (26, 170, 258–266). One reason for this is that self-rated health appears to be a simple, straightforward question. People are asked to rate their health on a five-point Likert-scale of poor, fair, good, very good, and excellent. Because five-point Likert-scales are non-equivalent across racial–ethnic groups and tend to elicit REM response styles, questions have been raised about the equivalence and validity of self-rated health across racial–ethnic groups.

An enormous number of studies [e.g., Ref. (26, 170, 266–276)] have found that Latinos (primarily) and Asians (and to a lesser extent, African-Americans as well) tend to rate their health as fair or poor despite health indicators to the contrary, and seem reluctant to rate their health as very good or excellent. This is most striking by language: Latinos and Asians who answer the self-rated health question in their native languages give significantly poorer ratings than their cohorts who answer in English [(259, 268–276); Table 7]. For example, Latinos answering the question in Spanish are seven times more likely than whites to report fair/poor health, whereas those answering in English are only twice as likely to do so (276). This has raised questions about whether the words “very good” and “excellent” have exact equivalents in other languages (259, 268). Moreover, self-rated health among Latinos and Asians also varies with nativity, years of US residence, acculturation (measured by acculturation scales), education, and income (26, 170, 258–276). Consequently, it is unclear if Latino and Asian disparities in self-rated health reflect lack of translation-equivalence, or the lower SES associated with low-English fluency (Table 7), or differences in their responses to Likert-scales (26, 170, 258–277).

Some have suggested that REMs’ fair/poor self-rated health and disparities in self-rated health reflect a cultural response-style specific to the self-rated health question, and that this style is a function of Collectivism (265, 277). White (and western-European) health questions presume that each person experiences him/herself in an Individualistic framework as an autonomous entity that one can reflect on to make statements about health, behaviors, and emotions (278). If people do not experience themselves in this manner, if they instead are Collectivists who are deeply intertwined with their families, how do they answer the self-rated health question? To whom do they refer? As Shweder and Sullivan [(278), p. 507] noted,

“in standard questions such as ‘How would you rate your overall health?’ it is not just the interpretation of the words ‘health’ and ‘overall’ that is problematic. The meaning of ‘your’ presents some fascinating problems as well. It is a plausible hypothesis that individuals in some ethnic groups are less willing to state that they are in excellent health or are *less able to experience themselves* in excellent health when other members of the family are suffering; new research is needed on cultural variations in the degree to which personal health and collective health are experienced as separate issues” (italics in the original).

#### Item order and improving equivalence of self-rated health

To complicate matters, Latino (in particular) and Asian self-rated health vary with where the question appears, i.e., with item order (258, 279). The normative practice in epidemiologic surveys is to place the self-rated health question before questions about specific health conditions (i.e., health without a context) to remove context effects. However, when asked to rate their health after (instead of before) answering questions about health conditions, Latinos (in particular) report better (more positive) health, whereas order has no effect on whites; this is especially the case for Spanish-speaking Latinos (258, 279). Such data suggest that rating one’s health without a context may be difficult for REMs, perhaps because of high Collectivism. Thus, some have empirically demonstrated and subsequently suggested that self-rated health should be asked after questions on specific health conditions to increase the equivalence and the validity of self-rated health across racial–ethnic groups (258, 279).

#### Socially desirable responding to scaled items

Socially desirable responding is the tendency to provide answers that one assumes to be consistent with social norms and expectations (desirable) irrespective of their veracity (280, 281). REMs exhibit significantly higher socially desirable responding than whites on Likert-scaled and yes/no-scaled items, and these racial–ethnic differences often are large, i.e., effect sizes ranging from *d* = 0.37 to 1.04 (280, 281). This socially desirable responding manifests as REM denial and under-reporting of the frequency of undesirable behaviors such as cigarette smoking (282–284), and as over-reporting the frequency of desirable behaviors such as cancer screening (285–288). For example, in the NHANES-III (in-person health survey with biologic measures taken), African-American cotinine-determined smokers were (OR) 4–9 times more likely than whites to deny smoking, with 68% of cotinine-determined Black-women smokers self-reporting non-smoking (282). Socially desirable responding is highest in household (in-person interviews), next highest in RDDTS, and lowest in anonymous mail or written surveys (281, 289). This has raised questions about the validity of racial–ethnic minority responses to scaled items in household interviews such as the NHIS and NHANES (281, 282).

African-American socially desirable responding has been theorized to reflect distrust of researchers, i.e., fear of the possible racist uses of their health data (30, 280, 281). This explanation is consistent with the finding that African-Americans omit significantly more items in health surveys and interviews than whites and other REMs as well (280, 281). Socially desirable responding among Latinos and Asians has been theorized to reflect Collectivism and its need to being viewed as conforming to others’ expectations (280, 281). Irrespective of its source, high socially desirable responding among REMs, like the response styles, undermines the validity of REM health data; can be misinterpreted as substantive group differences or the lack of them; and highlights the non-equivalence of Likert-scaled and of yes/no-scaled items across racial–ethnic groups (280, 281).

#### Reducing socially desirable responding

Many have suggested using social-desirability scales to detect and control for socially desirable responding (280, 281). The most widely used scales are the *Balanced Inventory of Desirable Responding* (290), and the *Marlow–Crowne Social Desirability Scale* (291, 292), the latter available in English and Spanish (293). These scales should be used with caution in light of ongoing debate regarding their validity (280, 281), and the fact that they contain Likert-scaled items. Alternatively, others have highlighted the benefits of cognitive-pretesting of surveys and interviews to improve item and scalar equivalence for diverse groups (231–233, 294, 295). Cognitive-pretesting involves verbal probing and interviewing of participants from diverse populations to assure that items and scaling are understood in the manner that researchers intend. Such techniques are simple (e.g., people read items and scaling aloud and talk about what they are thinking) and are well-established procedures for increasing both scalar and item equivalence across racial–ethnic and SES groups (295).

## Summary and Conclusion

This paper presented a review of the problem of measurement non-equivalence in epidemiologic research on racial–ethnic health disparities and provided concrete suggestions for improving sample, method, and scalar equivalence. Because our focus was delineating and illuminating non-equivalence and then suggesting strategies to improve equivalence, comprehensive reviews of topics (e.g., cultural variables, segregation) with all inconsistent findings reported were not presented. Although providing comprehensive, topical literature reviews is beyond the scope and purpose of this paper, the absence of those nonetheless is a limitation. Likewise, some variables that are highly relevant to racial–ethnic health disparities and therefore should be measured and controlled in studies could not be discussed due to space limitations – e.g., racial–ethnic discrimination (30, 42).

Moreover, due to space limitations, other important types of measurement equivalence (e.g., item, construct, translation, impact, etc.) could not be addressed, item equivalence in particular. Item equivalence refers to the extent to which the items in surveys and interviews are understood to mean the same thing by all social status groups; it is the extent to which the experiences (e.g., sadness), objects (e.g., cigars), and behaviors (e.g., smoking, vigorous physical activity, fruit/vegetable consumption) have the same referents for everyone (296–298). Although we could not cover the multitude of issues surrounding item equivalence, we highlight here that many of the ostensibly simple, straightforward questions in health surveys and interviews do not mean the same thing to diverse racial–ethnic groups, and have been demonstrated to be non-equivalent across those groups.

For example, when asked about ever and frequency of cigar use, REMs (youth in particular) often do not interpret the word “cigar” in the manner that researchers intend. Hence they significantly under-report cigar use unless specific cigar brand names are used (299–301). REMs’ self-reported cigar smoking nearly doubles when brand names are used (299), with the greatest increases found among African-Americans (300). Similar problems of the non-equivalence of items across race–ethnicity have been found for ostensibly simple questions about physical activity (302–304) and physical functioning (305, 306), and for a variety of other questions as well (225, 228, 307). Thus, the problems of non-equivalence across racial–ethnic groups extend well beyond what we could address here, and hence this presentation is limited in scope.

Likewise, due to space limitations, some relevant topics were not discussed. Specifically, Native Americans were included in the data on REMs presented here and were mentioned specifically, but studies of their health disparities were not separately addressed. In addition, propensity-scoring has been suggested as one possible solution to problems of equivalence (308) but was not addressed. Moreover, some forms of equivalence may be more important than others in specific types of health disparities research and with specific REMs but this issue could not be addressed here. Finally, some of our suggested measurement strategies for improving equivalence might be inter-related – e.g., measures of wealth might be correlated with measures of segregation and with prevalence of non-English proficiency, and REM response styles might be more prevalent among non-English speakers or in segregated communities. If and how some of these measures might interact is by and large unknown at present, and discussion of the few known interactions is beyond the purpose and scope of this paper. We recommend that researchers examine potential interactions among these measures just as they do among more normative measures.

Despite these limitations, issues of sample, method, and scalar non-equivalence were described in detail with explanations and examples presented as well. Likewise, a wide variety of concrete, simple strategies for improving measurement equivalence were provided to enhance epidemiologic studies of racial–ethnic health disparities. The challenge for epidemiologists is to cease using non-equivalent measures and methods, begin using alternatives that are equivalent across racial–ethnic groups, and define measurement equivalence as the gold standard in health disparities research. Doing so is critical because epidemiologic data on health disparities determine population forecasts, guide resource allocation, and shape efforts to reduce health disparities (13, 14).

## Conflict of Interest Statement

The authors declare that the research was conducted in the absence of any commercial or financial relationships that could be construed as a potential conflict of interest.
